# Modulation of metal-insulator transitions of NdNiO_3_/LaNiO_3_/NdNiO_3_ trilayers via thickness control of the LaNiO_3_ layer

**DOI:** 10.1038/s41598-019-56744-w

**Published:** 2019-12-27

**Authors:** Tai Nguyen, Van Hien Hoang, Tae-Yeong Koo, Nam-Suk Lee, Heon-Jung Kim

**Affiliations:** 1 Department of Physics, College of Natural and Life Science, Daegu University, Gyeongbuk, 38453 Republic of Korea; 2 Pohang Acceleration Laboratory (PAL) and X-ray Free Electron Laboratory (XFEL), Pohang University of Science and Technology (POSTECH), Pohang, 37673 Republic of Korea; 3 National Institute for Nanomaterials Technology (NINT), Pohang University of Science and Technology (POSTECH), Pohang, 37673 Republic of Korea; 4 Department of Materials-Energy Science and Engineering, College of Engineering, Daegu University, Gyeongbuk, 38453 Republic of Korea

**Keywords:** Materials science, Nanoscience and technology, Physics

## Abstract

Over the last few decades, manipulating the metal-insulator (MI) transition in perovskite oxides (ABO_3_) via an external control parameter has been attempted for practical purposes, but with limited success. The substitution of A-site cations is the most widely used technique to tune the MI transition. However, this method introduces unintended disorder, blurring the intrinsic properties. The present study reports the modulation of MI transitions in [10 nm-NdNiO_3_/t-LaNiO_3_/10 nm-NdNiO_3_/SrTiO_3_ (100)] trilayers (t = 5, 7, 10, and 20 nm) via the control of the LaNiO_3_ thickness. Upon an increase in the thickness of the LaNiO_3_ layer, the MI transition temperature undergoes a systematic decrease, demonstrating that bond disproportionation, the MI, and antiferromagnetic transitions are modulated by the LaNiO_3_ thickness. Because the bandwidth and the MI transition are determined by the Ni-O-Ni bond angle, this unexpected behavior suggests the transfer of the bond angle from the lower layer into the upper. The bond-angle transfer eventually induces a structural change of the orthorhombic structure of the middle LaNiO_3_ layer to match the structure of the bottom and the top NdNiO_3_, as evidenced by transmission electron microscopy. This engineering layer sequence opens a novel pathway to the manipulation of the key properties of oxide nickelates, such as the bond disproportionation, the MI transition, and unconventional antiferromagnetism with no impact of disorder.

## Introduction

Understanding and controlling the charge, spin and orbital orders are at the center of modern condensed matter physics and materials science^1–20^. In addition to high-*T*_*C*_ cuprates and manganites, the charge-transfer nickelate *RE*NiO_3_ (*RE* = rare earth) is a promising system with which to design emergent phenomena and novel functionalities based on heteroepitaxy. *RE*NiO_3_ possesses a rich phase diagram exhibiting a metal-insulator (MI) transition, antiferromagnetism with an unconventional ordering vector, and charge and bond disproportionation^4–24^. Bulk LaNiO_3_ (LNO) is the only member which is paramagnetic and metallic down to 0.4 K with no spin, charge, or bond ordering^25^. LNO has a rhombohedral structure with the R-3c space group. On the other hand, *RE*NiO_3_ is orthorhombic with the Pbnm space group in a high-temperature metallic phase. In a low-temperature insulating phase, its structure becomes monoclinic with P2_1_/n. As *RE*NiO_3_ is well described as a pseudo-cubic perovskite with tilts^25^, its electronic structure has been considered mainly to have the 3d^7^ configuration (Ni^3+^), consisting of the fully occupied triply degenerate *t*_*2g*_ and the singly occupied doubly degenerate *e*_*g*_.

This conventional view, however, is challenged by recent works^26–31^, which suggest that Ni has a valence closer to 2+ with holes in the oxygen 2p band due to negative charge transfer energy. Thus, the ground state of the paramagnetic state is more adequately described as a ${d}^{8}\underline{L}$ configuration (where $\underline{L}$ denotes a ligand hole). The negative charge transfer energy yields the MI transition in the form of bond disproportionation order. In the low-temperature insulating phase, self-doped oxygen 2p holes are coupled to alternating Ni sites, forming shrunken ${d}^{8}{\underline{L}}^{2}$ and an enlarged ${d}^{8}{\underline{L}}^{0}$ NiO_6_ octahedral. Strong antiferromagnetic coupling between the ligand and the Ni holes in ${d}^{8}{\underline{L}}^{2}$ cancels the magnetic moments, resulting in S = 0, while two *e*_*g*_ spins in ${d}^{8}{\underline{L}}^{0}$ lead to the triplet state with S = 1. This picture is quite consistent with charge and bond disproportionation and the unusual magnetic structure in the antiferromagnetic state.

The radius of the *RE* ion is the primary factor inducing different bond disproportionation properties because it affects the Ni-O-Ni bond angle, which determines the bandwidth and the metal-insulator transition temperature, T_MI_. Whereas LNO clearly does not undergo instability of the bond disproportionation order, other members of *RE*NiO_3_ are on the verge of this instability, undergoing a temperature-dependent transition into an antiferromagnetic insulating phase. Previously, the replacement of the smaller *RE* ions with larger La was considered to be an effective route to tune the strength of the bond disproportionation order^25,32^. However, random disorder in the *RE* sites can easily destroy an alternating ordering pattern of bond disproportionation, rapidly suppressing the MI transition temperature T_MI_ and the ordering strength.

To understand the intrinsic nature of bond disproportionation and unconventional antiferromagnetism, another method that can still modulate the ordering strength while not being influenced by disorder is necessary. In fact, oxygen octahedral tilts (rotation), which determine the degree of Ni-O hybridization, can be transmitted from the lower layer to the upper layer in perovskite heterostructures^33–35^. This transmission arises due to the geometrical constraint of the maintenance of the oxygen octahedral connectivity, causing the oxygen octahedral tilt to be more effectively enhanced when one layer is sandwiched by lower and upper layers with different tilts. Indeed, LNO, which has a tilt different from those of other nickelates, was predicted to be quite susceptible to different oxygen octahedral tilts, making it capable of transforming into other phases with different space groups, particularly in thin-film forms^36^. The present study finds that LNO on NdNiO_3_ (NNO) behaves as bulk NNO while NNO on LNO acts as bulk LNO. This fairly surprising result is likely caused by the influence of the lower layer on the oxygen octahedral tilt of the upper layer. Such an outcome is closely correlated with the other observation that LNO has an orthorhombic structure when it is sandwiched by NNO layers, as noted in transmission electron microscopy images. On the other hand, the single LNO layer on SrTiO_3_ (100) has a cubic structure. Because oxygen octahedral coupling at the interface usually decays by a few nanometers, the present results imply the triggering of a structural change of the entire layer via oxygen octahedral coupling at the interface. These structural characteristics fundamentally change the electrical transport properties and presumably the bond disproportionation.

We investigated NNO/LNO/NNO trilayers with the thicknesses of the bottom and top layers fixed at ~10 nm but with the thickness of the middle LNO layer varying from 5 nm to 20 nm. Upon increasing the LNO thickness, T_MI_ decreases with concomitant decreases of the residual resistivity. This suggests structural coupling between the NNO layers and the middle LNO, which weakens both the MI transition and the bond disproportionation order. It appears that through this coupling, the bond angle of the bottom NNO (the middle LNO) layer is transmitted to the middle LNO (the top NNO) layer. In heterostructures, two tendencies are competing. On the one hand, the geometrical connectivity restricts the Ni-O-Ni bond angle such that the bond angle of the middle LNO follows that of the bottom NNO. On the other, LNO tends to have a larger bond angle, as in the bulk, due to the larger ionic size of La. The former (latter) effect is stronger (weaker) when the middle LNO is thin. Hence, the thickness of the LNO layer becomes a control knob with which to tune the average bond angle of the entire trilayer. Unlike the substitution of *RE* site ions, this nanoscale approach based on a heterostructure tailors the strength of the bond disproportionation order and the MI and antiferromagnetic transitions, completely excluding the effects of random disorder.

## Methods and Materials

The LNO and NNO targets were fabricated via a sol-gel route. All films in this study were synthesized by means of pulsed laser deposition (PLD) at a wavelength of 248 nm. The optimal conditions for growing LNO and NNO thin films were presented in a previous report^37^. The LNO and NNO layers were synthesized at an oxygen partial pressure of 0.1 Torr with an energy density level of 1.0 J/cm^2^ and substrate temperature of 650 °C. Following this recipe, we deposited epitaxial trilayers of [10 nm-NNO/t-LNO/10 nm-NNO] with t = 5, 7, 10, and 20 nm (here, t refers to the thickness of the LNO layers) and bilayers consisting of LNO and NNO with equal thicknesses on SrTiO_3_ (STO) (001) substrates (Shinkosha, Japan). In the trilayers, the bottom NNO layer acts as a buffer. LNO and NNO single-layer films were also synthesized under identical conditions for comparison. The total thickness of both the LNO and NNO single-layer films and the bilayers was fixed at approximately 30 nm. To evaluate crystallinity of the thin films, we took synchrotron x-ray diffraction measurements at an energy level of 11.3 keV at the 3 A beamline in the Pohang Accelerator Laboratory (PAL). Reciprocal space mapping was measured on the 5 nm and 20 nm LNO trilayers by scanning around the (103) reflections of STO. We investigated the film morphology using atomic force microscopy (AFM) and the local structure of the trilayers with a transmission electron microscope (TEM, Cs-corrected, JEOL JEM-2100F). The thickness of the films was estimated via x-ray reflectivity (XRR) and TEM. Subsequently, measurements of the temperature-dependent sheet resistance were taken from 300 K to 2 K in a physical property measurement system (PPMS, Quantum Design Inc.) using the van der Pauw method.

## Results and Discussion

**Figure 1:**
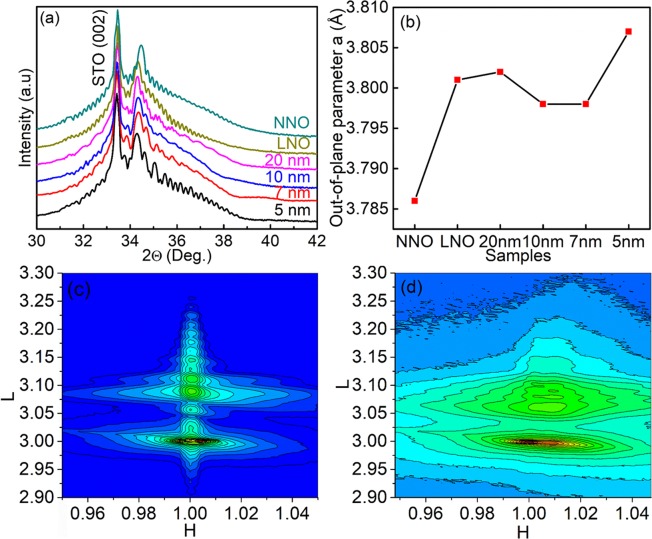
(**a**) *X*-ray diffraction patterns of single NNO and LNO layers and trilayers with different thicknesses of the LNO layer (5, 7, 10, 20 nm) in the vicinity of the (002) reflection. (**b**) Out-of-plane (*oop*) parameters a_*oop*_ for LNO and NNO single layers and trilayers with LNO layers (5, 7, 10, 20 nm). Reciprocal space mapping around the (103) reflections of STO for (**c**) the 5 nm LNO trilayer and (**d**) the 20 nm LNO trilayer.

Figure 1(a) presents the synchrotron x-ray diffraction (XRD) results that reveal the film quality. Films are oriented toward the c-axis without an impurity phase. The XRD patterns show periodic oscillations of the intensity, referred to as the Kiessig fringes. It should be noted that the (002) peaks of the trilayers are located closer to that of the single LNO film than to that of the single NNO film. Figure 1(b) summarizes the out-of-plane lattice parameters of the trilayer and single-layer films as deduced from the XRD results. The out-of-plane lattice parameters of the trilayers are closer to that of the single LNO film than they are to that of the single NNO film. The Kiessig fringes in the XRD patterns and the small root-mean-square (RMS) roughness estimated from the AFM images (Fig. S1) confirm the high quality of the films, with smooth interfaces and surfaces. The RMS roughness values were estimated to be 0.753, 0.953, 0.391, 1.26, 0.344, and 0.528 nm for the 5 nm, 7 nm, 10 nm, 20 nm trilayers, the LNO, and the NNO single layers, respectively. We did not observe any cracks or pinholes in the AFM images. Figure 1(c,d) present reciprocal space maps (RSMs) near the (103) reflections of the 5 nm and 20 nm trilayers, respectively. While the 5 nm trilayer is fully strained, the strain of the 20 nm trilayer is partially relaxed.

**Figure 2:**
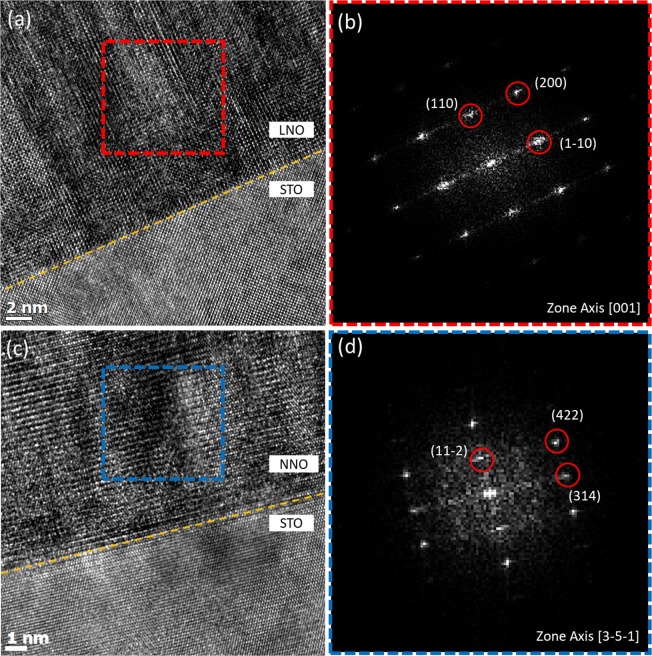
TEM images of (**a**) a single 30 nm LNO layer and (**c**) a single 30 nm NNO layer on (100) STO substrates. The fast Fourier transform (FFT) patterns of the real-space images of LNO (**b**) and NNO (**d**) in the selected area denoted by the dotted red and blue rectangles are shown in (**a**,**c**), respectively. While the single LNO layer has a cubic structure, the single NNO layer possesses an orthorhombic structure.

**Figure 3:**
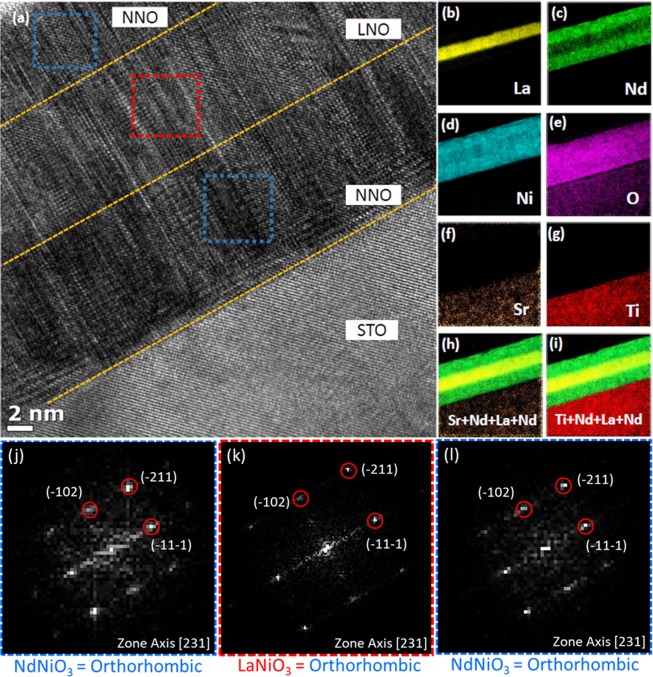
(**a**) TEM image of the 10 nm-NNO/10 nm-LNO/10 nm-NNO heterostructure on a SrTiO_3_ substrate. (**b**–**i**) EFTEM elemental mapping outcomes of La, Nd, Ni, O, Sr, Ti, Ti + Nd + La + Nd, and Sr + Nd + La + Nd, respectively. FFT patterns of the real-space images for the top NNO layer (**j**), the middle LNO (**k**) and the bottom NNO layer **(l**) in the selected area denoted by the dotted blue, red, and blue rectangles in (**a**), respectively.

Although the synchrotron x-ray diffraction measurements and AFM images qualitatively confirmed the high quality of the interfaces, these probes are rather indirect characterization techniques for the interfaces here. Figures 2 and 3 display TEM results of the single layers and the 10 nm trilayer, respectively. As shown in Fig. 2(a,c), the STO substrate-film interfaces of the single layers are atomically abrupt. In addition, the fast Fourier transform (FFT) pattern of the real-space images, shown in Fig. 2(b,d), confirms the structures of the single layers; whereas the NNO single layer is orthorhombic, LNO is found to be cubic. For the single NNO, the structure of the film is identical to the bulk. In contrast, in the case of the single LNO, a structural change from rhombohedral to cubic occurs due to the influence of the STO substrate. In a cubic structure, LNO is expected to have a Ni-O-Ni bond angle larger than or comparable to that in the bulk case, implying metallic properties of the single LNO layer. As the structure of the single NNO does not change, it is also expected to exhibit electrical transport properties similar to those of the bulk NNO.

The TEM image of the trilayer [Fig. 3(a)] demonstrates that this trilayer is uniform, and the interface is coherent and atomically abrupt. It is important to note that the interface between the LNO and NNO layers is scarcely visible in the TEM image due to the small Z contrast between lanthanum and neodymium. With energy-filtered transmission electron microscopy (EFTEM) elemental mapping, the interface could be identified more clearly. Here spatial distributions of La, Nd, Ni, O, Sr, and Ti ions were acquired by detecting the La-*M*_4,5_, Nd-*M*_4,5_, Ni-*L*_2,3_, O-*K*, Sr-*M*_4,5_, and Ti-*L*_2,3_ edges, respectively. Figure 3(b–i) show the EFTEM elemental mapping outcomes of La, Nd, Ni, O, Sr, Ti, Ti + Nd + La + Nd, and Sr + Nd + La + Nd, respectively. All of the layers and interfaces are clearly visible. No significant diffusion of lanthanum into the NNO layers or neodymium into the LNO layer could be observed. Interesting features in the FFT pattern of the selected areas are shown in Fig. 3(j–l). While the bulk LNO is rhombohedral, the middle LNO layer in the trilayer has an orthorhombic structure, identical to the lower and upper NNO layers. This is evidence of the strong influence of the lower NNO layer on the upper LNO in the trilayer structure. This result differs remarkably from the structure of the single LNO layer, which is cubic. On the other hand, the single NNO layer is orthorhombic, as expected. The TEM results thus strongly suggest a Ni-O-Ni bond angle smaller in the middle LNO layer than in the single LNO. This also affects the metallicity, which is believed to be weaker in the trilayers than in the single LNO layer. We also confirm that all three layers in the trilayer have the same orthorhombic structures using high-angle annular dark-field scanning transmission electron microscopy (HAADF-STEM) images presented in the supplementary (Fig. S4).

**Figure 4:**
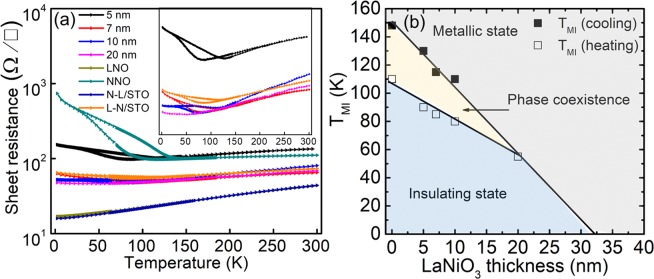
(**a**) Temperature dependence of sheet resistance for the LNO and NNO single layers, the trilayers, and bilayers. The inset compares the R-T curves of the trilayers and L-N/STO bilayer. (**b**) Transition temperatures of the NNO single layer and heterostructure trilayers as a function of LaNiO_3_ thickness. Lines are guides for the eyes.

Related to the structural characteristics observed above, another intriguing feature of the trilayer heterostructures is the electrical transport properties. Figure 4(a) presents the temperature dependence of the sheet resistance from 300 K to 2 K, which demonstrates the influence of the thickness of the LNO. Notably, the sheet resistance at T = 2 K of the trilayers is smaller than that of the single NNO film, decreasing with an increase in the thickness of the middle LNO layer. With a decrease of the temperature, all samples except LNO undergo MI transitions. In the metallic region (T > T_MI_), the sheet resistance decreases almost linearly with a decrease in the temperature. This suggests the dominant contribution of the electron-phonon scattering at high temperatures^32^. In the insulating region (T < T_MI_), the sheet resistance is prominently hysteretic.

In the van der Pauw geometry of a trilayer structure with distinct layers, the applied current is divided into three different layers, and only the partial current flows in each of the three layers. In such a case, the trilayer can be approximated as an effective circuit with parallel resistances. The sheet resistance of the trilayers can be expressed as the parallel sum of the layer resistances. If the NNO layers had bulk properties without any influence of the middle LNO, T_MI_ of the trilayers would be independent of the LNO thickness. This is in sharp contrast to the observations presented in Fig. 4(a), strongly suggesting that the distinction between the layers is not clear and that uniform current flows in the trilayer. This is due to the influence of the bottom NNO on the middle LNO layer, which changes the characteristics of the LNO to those of NNO. In fact, this behavior is quite consistent with the TEM findings in that the LNO has an orthorhombic structure identical to that of the NNO layers in the trilayer.

The inset of Fig. 4(a) displays the metal-insulator transition of the trilayers in detail. Here, we define the MI transition temperature, T_MI_, as the inflection point of the curves^38^. The T_MI_ values of trilayers extracted from this method are presented in Fig. 4(b). The T_MI_ value decreases as the thickness of the LNO layer increases. T_MI_ is expected to become zero at the critical thickness of about 32 nm. It is important to note that the hysteresis of the 20 nm trilayer nearly vanishes, though it exists in the other trilayers. Hysteresis in the resistance is a signature of the first-order MI transition, signifying the coexistence of different domains with different bond disproportionation characteristics^39,40^. The difference in the T_MI_ value of the cooling and heating curves is as large as 40 K. The absence of hysteresis in the 20 nm trilayer indicates the change in the nature of the phase transition; in this case, a change occurs from the first-order transition to the second-order transition or the emergence of localization. If it is localization, the nature of the insulating phase in the 20 nm trilayer will differ from that in the other trilayers.

The dependence of T_MI_ on the thickness of the middle LNO cannot be accounted for by the strain effect. In fact, according to the RSM measurements, the strain of the 20 nm trilayer is partially released, whereas the 5 nm trilayer is fully strained. Despite such contrasting strain states, they both show MI transitions that scale quite well with the LaNiO_3_ thickness. Moreover, the STO substrate, which exerts tensile stress on the nickelate layers, is known to favor octahedral breathing distortion. This distortion is known to stabilize the bond disproportionation order^41^. Thus, if the misfit strain were the main factor, T_MI_ should not decrease because the bond disproportionation becomes stronger. This strongly implies that there is another factor that controls T_MI_.

Instead of the strain effect, we found that the layer sequence influences the electrical transport properties more significantly. We synthesized two bilayers with equal thicknesses - one with NNO on top of LNO (N-L/STO) and the other with LNO on NNO (L-N/STO). Surprisingly, the N-L/STO sample is metallic down to 2 K with no sign of a phase transition, whereas L-N/STO exhibits upturns with hysteresis as in the single NNO and trilayer structures [Fig. 3(a)]. This difference implies the influence of the lower layer on the upper. While the lower layer has electrical properties similar to those of the corresponding bulk, the upper layer electrical transport properties that match those of the lower layer. In N-L/STO, the upper NNO has metallic properties identical to those of the bulk LNO. On the other hand, in L-N/STO, the upper LNO has the electrical characteristics similar to those of the bulk NNO.

Recently, the possibility of manipulating the oxygen network on the unit-cell level was reported^33,34^. In these reports, oxygen octahedral rotation was demonstrated to be transmitted from the lower layer to the upper when a suitable substrate or a buffer layer was used. Such oxygen octahedral coupling is so effective that even a buffer with the thickness of a single unit cell can change the metal-oxygen-metal bond angle of the subsequent layers, stabilizing bond angles even when inaccessible in the bulk. Our results can be understood by considering this mechanism, i.e., successive transfer of the Ni-O-Ni bond angle from the lower layer to the upper. Bulk LNO is rhombohedral (space group R-3c) with *a*^*−*^*a*^*−*^*a*^*−*^ octahedral tilts in Glazer’s notation^42^ while bulk NNO is orthorhombic (space group Pbnm) with *a*^*−*^*b*^+^*a*^*−*^. As only the tilt around the pseudocubic [010] direction is different, a LNO layer a few unit cells thick with the *a*^*−*^*b*^+^*a*^*−*^ tilt on the bulk NNO layer can have energy lower than a LNO layer a few unit cells thick with *a*^*−*^*a*^*−*^*a*^*−*^. As the total energy of the LNO layer is a function of the tilt angle, it is expected that the bond angle approaches the bulk value with an increase in the layer thickness. Therefore, the thickness of the LNO layer determines the average bond angle. Indeed, a change in the bond angle with the layer thickness was observed in the SrRuO_3_/Ca_0.5_Sr_0.5_TiO_3_ system^34^. Bond disproportionation, MI, and antiferromagnetic transitions can be tailored by changing the bond angle transmitted from the lower layer through oxygen octahedral coupling. The average bond angle is determined by the LNO thickness.

It is noted that T_MI_ of the single NNO layer is 150 K, which lower than the bulk value by 50 K. This suggests possible existence of oxygen deficiency in the present samples. According to the studies of electron doping effects on the bulk NNO^43,44^, the decrease of T_MI_ by 50 K corresponds to ~5% electron doping. If electron were doped entirely due to the formation of oxygen deficiency, the value of δ in the chemical formula NdNiO_3-δ_ is estimated to be 0.025.

## Conclusion

In summary, we have studied the effect of the LaNiO_3_ thickness on the structural and electrical transport properties of NdNiO_3_/LaNiO_3_/NdNiO_3_/SrTiO_3_(100) trilayer heterostructures with different LaNiO_3_ thicknesses of 5, 7, 10, and 20 nm and with the bottom and top NdNiO_3_ thicknesses fixed at 10 nm. The sheet resistance versus the temperature of the trilayers has revealed that the metal-insulator transition temperature T_MI_ decreases as the thickness of the LaNiO_3_ layer increases with a concomitant decrease of the sheet resistance at 2 K. This decrease of T_MI_ and the concomitant reduction of the sheet resistance at 2 K can be understood as occurring within a framework of oxygen octahedral coupling. Indeed, TEM was used to observe the orthorhombic structure of the middle LaNiO_3_ in the trilayer, demonstrating that all three layers have an identical orthorhombic structure. To clarify the scenario of oxygen octahedral coupling further, we also carried out electrical transport measurements on two bilayers, i.e., NdNiO_3_/LaNiO_3_/SrTiO_3_(100) and LaNiO_3_/NdNiO_3_/SrTiO_3_(100) films. While the NdNiO_3_/LaNiO_3_/SrTiO_3_(100) bilayer remains metallic down to 2 K, the LaNiO_3_/NdNiO_3_/SrTiO_3_(100) bilayer undergoes the metal-insulator transition between 85 K and 125 K. This result suggests that while the lower layer has electrical properties similar to those of the corresponding bulk, the upper layer follows the electrical transport properties of the lower layer. Our study demonstrates that trilayer heterostructures with the NdNiO_3_/LaNiO_3_/NdNiO_3_/SrTiO_3_(100) sequence allow for systematic variations of T_MI_ and thus a change of the bond disproportionation strength via control of the LaNiO_3_ thickness with no impact on the degree of disorder. The nanoscale approach using layer engineering paves the way toward a better understanding of the intrinsic nature of the metal-insulator transition, bond disproportionation, and unconventional antiferromagnetism of perovskite nickelates.

## Supplementary information

Supplementary Information.
