# Supplementary material for: Modulation of metal-insulator transitions of NdNiO3/LaNiO3/NdNiO3 trilayers via thickness control of the LaNiO3 layer
Source: Sci Rep. 2019 Dec 27;9:20145. doi: 10.1038/s41598-019-56744-w (PMC6934756; doi:10.1038/s41598-019-56744-w)
Supplement: Supplementary file 1 — Supplementary Information. [file 41598_2019_56744_MOESM1_ESM.pdf]

SUPPORTING INFORMATION

Modulation of metal-insulator transitions of $NdNiO_3/LaNiO_3/NdNiO_3$ trilayers via thickness control of the $LaNiO_3$ layer

Tai Nguyen[1], Van Hien Hoang[1], Tae-Yeong Koo[2], Nam-Suk Lee[3,*] and Heon-Jung Kim[1,4,§]

[1]*Department of Physics, College of Natural and Life Science, Daegu University, Gyeongbuk 38453, Republic of Korea*

[2]*Pohang Acceleration Laboratory (PAL) and X-ray Free Electron Laboratory (XFEL), Pohang University of Science and Technology (POSTECH), Pohang, 37673, Republic of Korea*

[3]*National Institute for Nanomaterials Technology (NINT), Pohang University of Science and Technology (POSTECH), Pohang, 37673, Republic of Korea*

[4]*Department of Materials-Energy Science and Engineering, College of Engineering, Daegu University, Gyeongbuk 38453, Republic of Korea*

[*] nslee@postech.ac.kr

[§] hjkim76@daegu.ac.kr

The AFM images were obtained using the contact mode and scanning in lateral dimension of 5x5 μm^2, as shown in Fig. S1. The RMS roughness were estimated to be 0.753, 0.953, 0.391, 1.26, 0.344, and 0.528 nm for the 5nm, 7nm, 10nm, and 20nm trilayers and the LNO and NNO single layers, respectively.

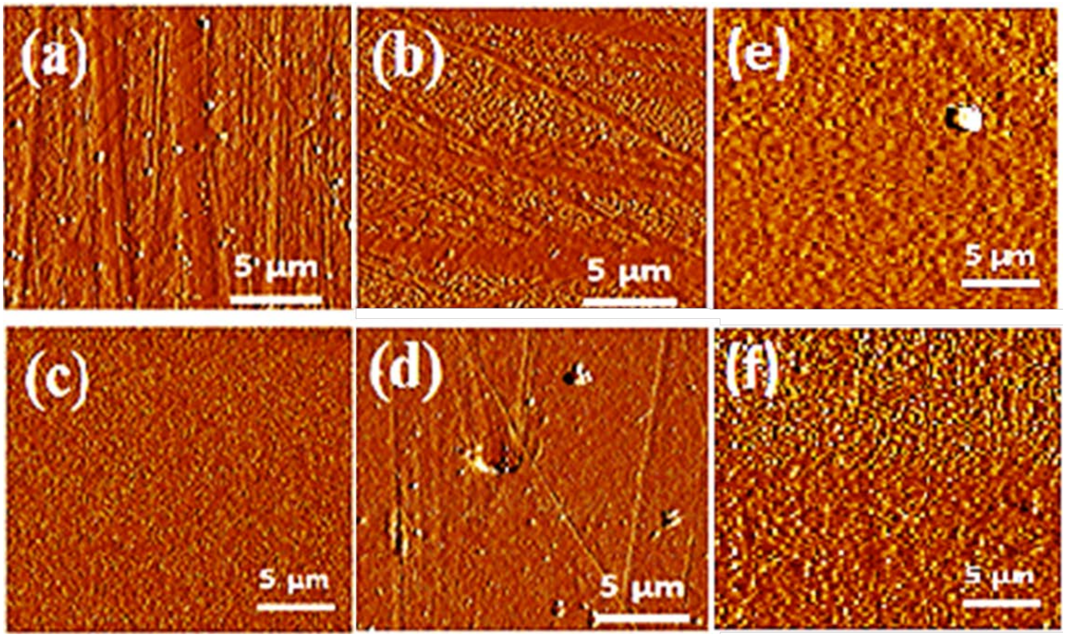

Figure S1: AFM images of trilayers with LNO thicknesses of (a) 5 nm, (b) 7 nm, (c) 10 nm, and (d) 20 nm. (e) and (f) show AFM images of LNO and NNO single layers, respectively.

Figure S2 and Figure S3 show transmission electron microscopy images (TEM) and energy-filtered transmission electron microscopy (EFTEM) elemental mapping for single 30nm LNO and single 30nm NNO thin films, respectively. For the EFTEM mapping, the spatial distributions of the La, Nd, Ni, O, Sr, and Ti ions were acquired by detecting the La-$M_{4,5}$, Nd-$M_{4,5}$, Ni-$L_{2,3}$, O-K, Sr-$M_{4,5}$, and Ti-$L_{2,3}$ edges, respectively. Figure S4 presents the high-angle annular dark-field scanning transmission electron microscopy (HAADF-STEM) images of the trilayer. No difference among the layers was observed in the real space images and the FFT patterns.

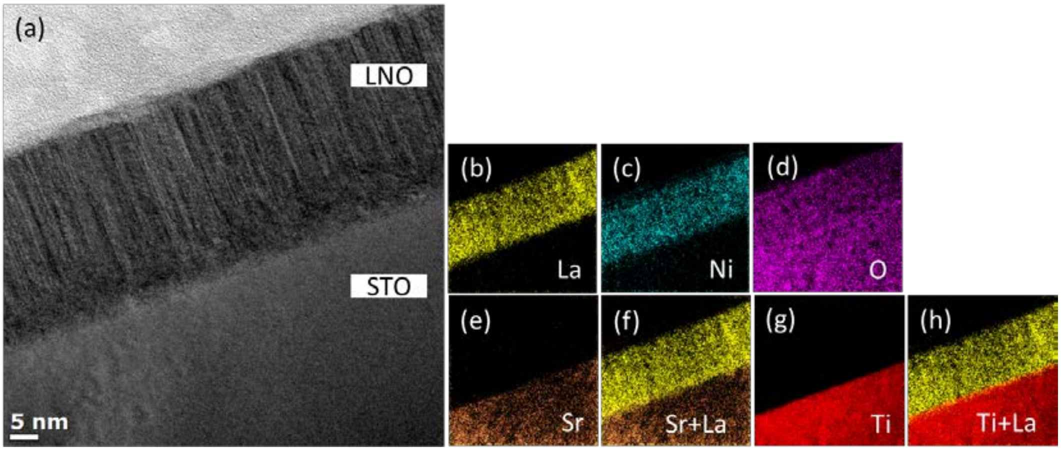

Figure S2: (a) TEM image of a single 30nm LNO thin film on a $SrTiO_3$ substrate. (b)-(h) EFTEM elemental mapping results of La, Ni, O, Sr, Sr+La, Ti, and Ti+La, respectively.

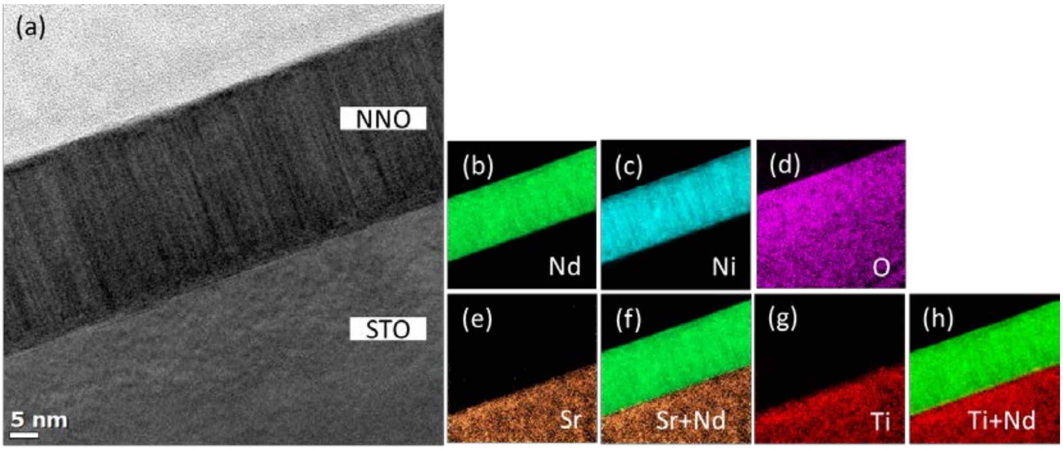

Figure S3: (a) TEM image of a single 30nm NNO thin film on a $SrTiO_3$ substrate. (b)-(h) EFTEM elemental mapping results of Nd, Ni, O, Sr, Sr+Nd, Ti, and Ti+Nd, respectively.

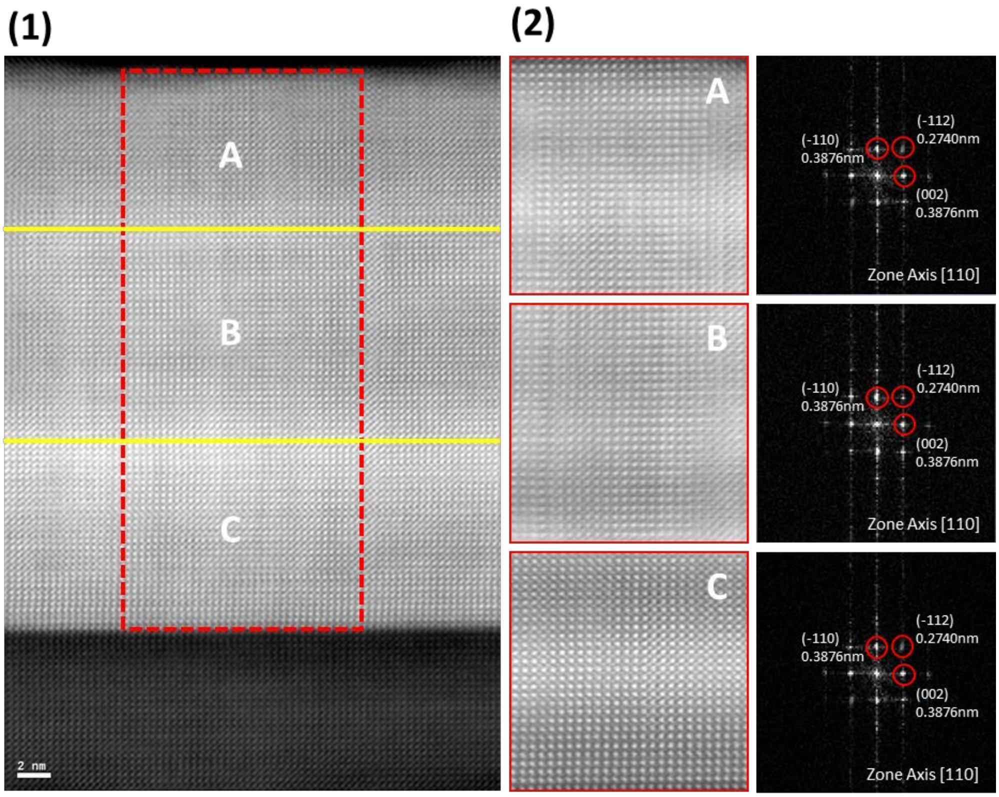

Figure S4: (1) The HAADF-STEM image of the 10nm-NNO/10nm-LNO/10nm-NNO heterostructure on a $SrTiO_3$ substrate, (2) The HAADF-STEM images and the FFT patterns of the A, B, C areas, respectively are given. Here A, B, C are corresponding to the top NNO (orthorhombic), the central LNO (orthorhombic), and the bottom NNO (orthorhombic) layers.
